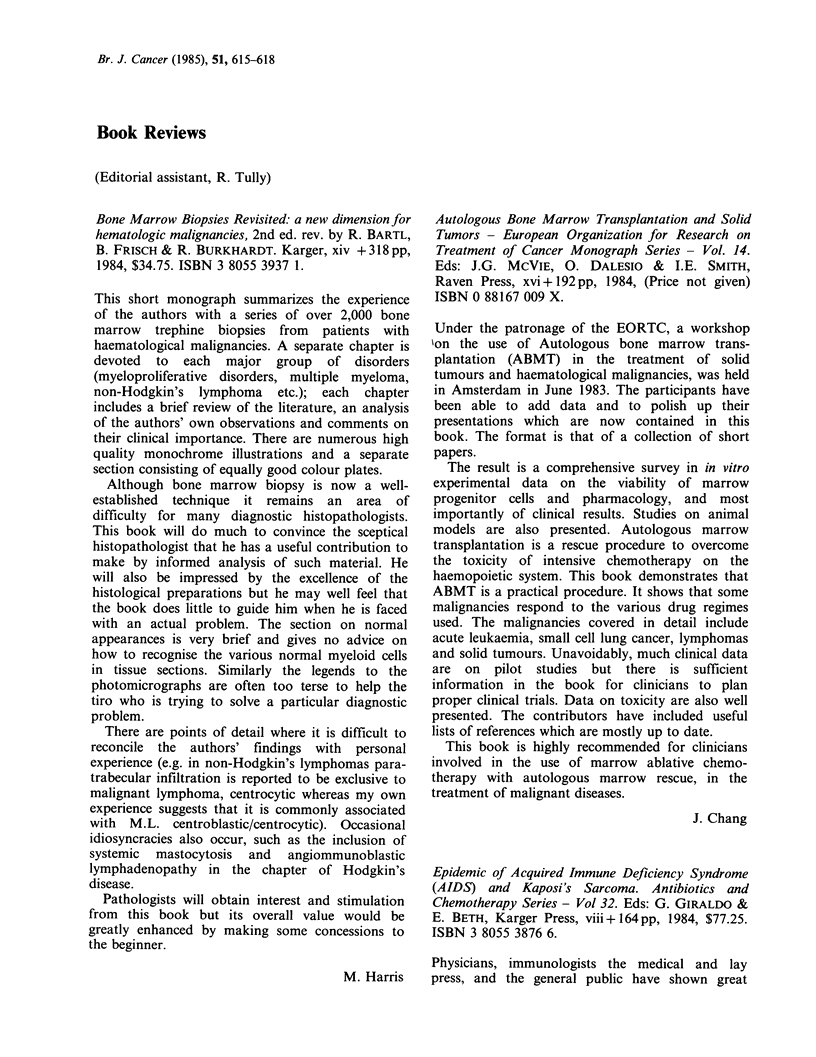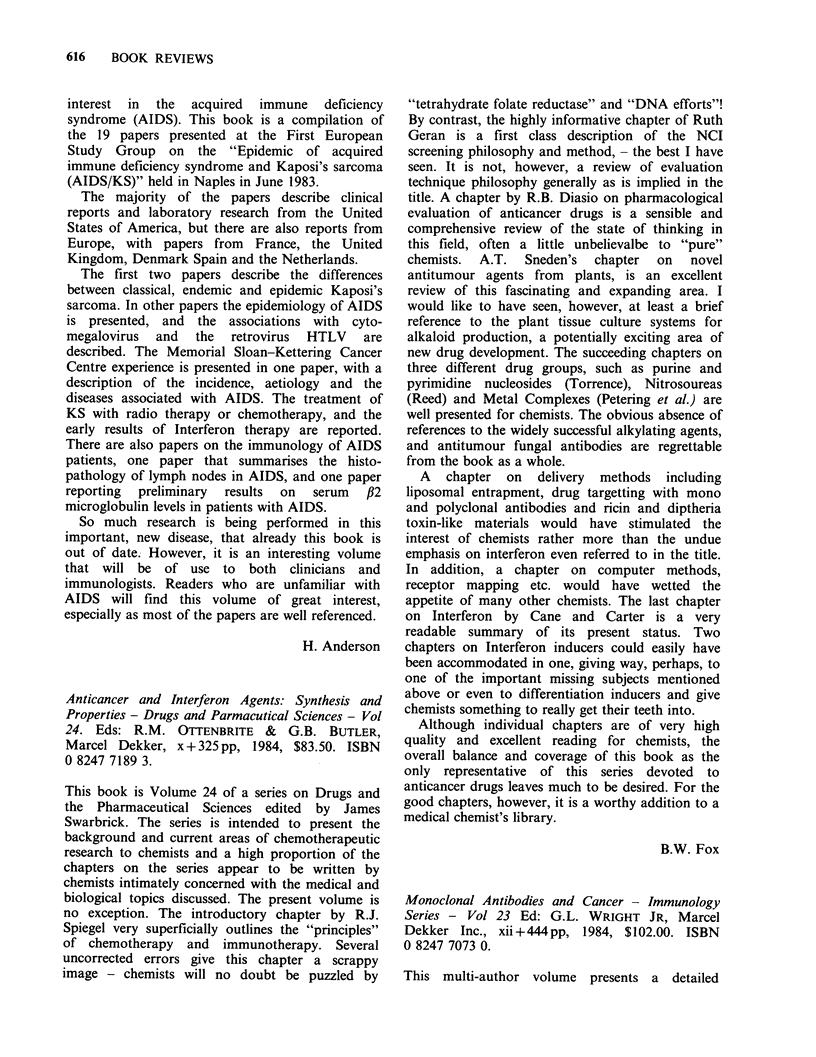# Epidemic of Acquired Immune Deficiency Syndrome (AIDS) and Kaposi's Sarcoma. Antibiotics and Chemotherapy Series - Vol. 32

**Published:** 1985-04

**Authors:** H. Anderson


*Epidemic of Acquired Immune Deficiency Syndrome (AIDS) and Kaposi's Sarcoma. Antibiotics and Chemotherapy Series – Vol 32.* Eds: G. GIRALDO & E. BETH, Karger Press, viii+164 pp, 1984, $77.25. ISBN 3 8055 3876 6.

Physicians, immunologists the medical and lay press, and the general public have shown great interest in the acquired immune deficiency syndrome (AIDS). This book is a compilation of the 19 papers presented at the First European Study Group on the "Epidemic of acquired immune deficiency syndrome and Kaposi's sarcoma (AIDS/KS)" held in Naples in June 1983.

The majority of the papers describe clinical reports and laboratory research from the United States of America, but there are also reports from Europe, with papers from France, the United Kingdom, Denmark Spain and the Netherlands.

The first two papers describe the differences between classical, endemic and epidemic Kaposi's sarcoma. In other papers the epidemiology of AIDS is presented, and the associations with cytomegalovirus and the retrovirus HTLV are described. The Memorial Sloan–Kettering Cancer Centre experience is presented in one paper, with a description of the incidence, aetiology and the diseases associated with AIDS. The treatment of KS with radio therapy or chemotherapy, and the early results of Interferon therapy are reported. There are also papers on the immunology of AIDS patients, one paper that summarises the histopathology of lymph nodes in AIDS, and one paper reporting preliminary results on serum $\beta 2$ microglobulin levels in patients with AIDS.

So much research is being performed in this important, new disease, that already this book is out of date. However, it is an interesting volume that will be of use to both clinicians and immunologists. Readers who are unfamiliar with AIDS will find this volume of great interest, especially as most of the papers are well referenced.

H. Anderson